# Continuous-Gum Work

**Published:** 1899-04

**Authors:** 


ARTICLE III.

### CONTINUOUS GUM WORK.

### A FEW METHODS OF MAKING FULL SETS

First Method.—After as perfect an impression of the mouth has been taken as it is possible to obtain, and the model and dies have been cast, a plate of No. 4 hard platinum (Ash's gauge) is swaged or struck up with a flanged edge all around, which serves as a support to the mineral body and makes it smooth and comfortable to the tissues of the mouth with which it comes in contact. This flanged edge is produced in the following manner: Before the zinc die is cast, a wall of wax is built up all around the outside of the model, up to where it is intended to bring the plate, and trimmed down with an inward slope towards the model in the form of a flat shelf, so that when the zinc die and lead counter are cast, the counter will have a convex ridge, the zinc a corresponding concavity, and consequently the edge of the plate will be turned over or flanged during the process of swaging or striking-up.

The same result can be obtained by soldering with fine gold a piece of thin platinum wire on the extreme edge of the plate, but it is very much more difficult to do this than to form the flanged edge in the way described.

After the plate has been swaged or struck-up, the teeth are backed with platinum and soldered to the plate with fine gold, or attached by means of a length of platinum wire, which is better, as the wire lends strength to the mineral body. It is then ready for the application of the body, which is mixed as thickly as it can be worked, packed in between the teeth with a spatula, built up on the labial side and contoured to the desired shape and thickness of the intended gum. It should be borne in mind that any shape or contour aimed at is made at this first stage with the body; the gum enamel is only a coloring agent, and should always be laid on thinly when it is applied. The palate should now be covered with the body, and the whole of the work allowed to dry naturally. When thoroughly dry, the case is ready for firing. In order to obtain the greatest possible amount of strength in continuous-gum work, the body and enamel should not be thoroughly fused or vitrified more than once.

The best method of doing the work throughout is as follows.

The first application of the body mentioned above, on an upper or lower case, is only fired sufficiently to solidify the material. This permits of any defects in the building-up or contour being readily seen and rectified.

After the second layer of body has been applied, the case is again put in the furnance, and the firing is carried to the biscuiting stage.

The first layer of gum enamel is then thinly applied and fired sufficiently to slightly glaze it, after which any desired alteration in color is made by the addition of a little more enamel if the shade be too pale, or by grinding off a little if too dark; the case is again placed in the furnace and finally fired until it is thoroughly fused.

The first three firings need careful watching to avoid vitrifying the materials. In the final firing the heat should be raised until a test portion of the body fuses and balls up, which should be laid on a small nickel tray against the front of the case, and when the glaze is seen to appear on the surface of the case it is sufficiently fused.

The floor of the muffle should be covered with a thin layer of investing material in the form of dry powder.

A few experiments will enable the worker to detect the appearance of the glaze and render it easy for him to determine when the firing is completed without the use of a test portion.

These remarks upon firing equally apply to the second and third methods of making full sets, which are given below.

Second Method.—The special feature about this method is the use of diatoric instead of plate teeth. After the platinum plate has been swaged or struck up as described above, the diatoric teeth are waxed to the plate in the same way as for a vulcanite case; three sections in plaster of paris are built around the model and waxed case, one in front of the six incisors, one facing the bicuspids and molars on the right side, and the third facing the same teeth on the left side—each up to within an eighth of an inch of the cutting edges and crowns of the teeth. Of these sections the front one should be made first, and when it has set it should be soaped. The two side sections can then be made from one mix of plaster. If holes be cut, one on the front and one at each side of the plaster model, before the sections are made, corresponding knobs will be formed on the inside of each section, which holes and knobs will serve to keep the sections in position.

The case is then soaped and a fourth section is bult up, on the top of the front and side sections and over the crowns and cutting edges of the teeth, which unites all and keeps them together.

The wax is now scalded out; the platinum base is pickled in nitric acid, to ensure the thorough removal of any particles of lead which may have adhered to it from the dies; the plaster sections are removed and lined with tissue paper (cigarette paper is the best for the purpose), the creases in which can be smoothed out with a camel-hair pencil dipped in vaseline. The tissue paper prevents the gum body sticking to the plaster sections during packing. The body is now mixed fairly thick and packed in with a fine spatula and camel-hair pencils until an exact representation of the wax is reproduced. The body is then applied to the palatal side of the platinum base, and the case is allowed to stand until it is thoroughly dry. This plan of drying naturally yields better results than drying and heating up in the furnace, preparatory to firing. When thoroughly dry, the plaster sections are removed and the case is taken off the model and fired, after every particle of body which may be found on the faces, crowns or cutting edges of the teeth has been carefully removed

It is possible after a little practice to make full sets with diatoric teeth, especially lower sets, without using plaster sections. This of course greatly simplifies the work, and admits of its being quickly done.

The following is a brief description of the manner in which such sets are made: After the plate is swaged or struck up, a layer of gum body is spread over the alveolar ridge, put in the furnace, slightly heated, removed, and, while warm, fresh body is added, and the teeth are placed in position one at time. The warm body absorbs the moisture from the fresh body, causes each tooth to remain in situ, and thus permits of all being quickly set up. After this is done, the case is put in the articulating frame, and when the bite has been obtained, any moisture in the body is driven out by slightly heating in the furnace. When cool, the palatal surface of the plate is covered with body, the case is allowed to dry naturally, and then sunk in dry investing material on a nickel tray ready for firing.

If care be taken in firing, as described above, no alteration, but such as can be easily rectified, will be found to have taken place in the position of the teeth.

Third Method.—There are perhaps more difficulties in attaching a vulcanite base to a full set continuous-gum facing than in doing any other kind of dental mechanical work, but with care and experience such dentures can be satisfactorily made in this way: A frame plate is waged or struck up to fit over the alveolar ridge and covering about half an inch of the palatine surface, to which small tags of platinum are soldered with fine gold, or loops may be made in the plate, with Peck's or Gartrell's loop punch, to afford a hold for the vulcanite. The teeth are then attached to the plate, just as on a full continuous-gum denture, a flat band or wire is fastened across the posterior end of the palate to the heel of the plate, to counteract warping which would otherwise take place, in a case of this kind, under the extreme heat to which it is subjected. The body and the enamel are then applied and fired, as already described under the first method.

In vulcanizing, the following precautions are necessary: The flask should be as small as can be used, the object being to employ the least possible amount of plaster of paris; the various stages of flasking should be carried out without any long intervals between them, for if the plaster be allowed to harden and become dry, the subsequent softening in the vulcanizer causes undue expansion, and small cracks are made in the continuous-gum facing. On no account should the case be vulcanized on the model, but sunk in plaster in the flask. When the model is just hard enough to receive the rubber, it should be packed and placed in boiling water before the flask is closed; dry heat should never be used in lieu of boiling water. Another fruitful cause of cracks in the continuous-gum facing is the contraction of the rubber while cooling, and for this reason it is always deemed advisable to make the rubber base as thin as possible, and to let the case remain in the vulcanizer until it is quite cold. When it is taken out of the flask it is placed in hot water for a few moments, and the plaster is removed from it with an old tooth brush.—*Ash's Quarterly*.

---

ARTICLE IV.

